# Short Peptide and Amino Acid-Based Supramolecular Ionogels and Eutectogels

**DOI:** 10.1002/chem.202400622

**Published:** 2024-06-10

**Authors:** Carina Esteves, Ana Cecília A. Roque

**Affiliations:** a Associate Laboratory i4HB – Institute for Health and Bioeconomy, NOVA School of Science and Technology, NOVA University of Lisbon, Campus da Caparica, 2829-516 Caparica (Portugal); b UCIBIO – Applied Molecular Biosciences Unit, Chemistry Department, NOVA School of Science and Technology, NOVA University of Lisbon, Campus da Caparica, 2829-516 Caparica (Portugal)

**Keywords:** supramolecular self-assembly, peptide, amino acid, ionic liquid, deep eutectic solvent

## Abstract

The capability of peptide and amino acid-based molecules to act as ionogelators and eutectogelators entrapping ionic liquids (ILs) and deep eutectic solvents (DESs) forming ionogels and eutectogels has gathered attention in recent decades. The self-assembly process, primarily driven by non-covalent interactions as hydrogen bonding, remains serendipitous in nature. This review provides a comprehensive and detailed report on self-assembly of unmodified and modified amino acids and peptides in the non-conventional solvents, ILs and DESs. Understanding these processes holds great promise for the development of innovative soft-materials, and to the progress of supramolecular systems in non-conventional solvent environments.

**Figure:**
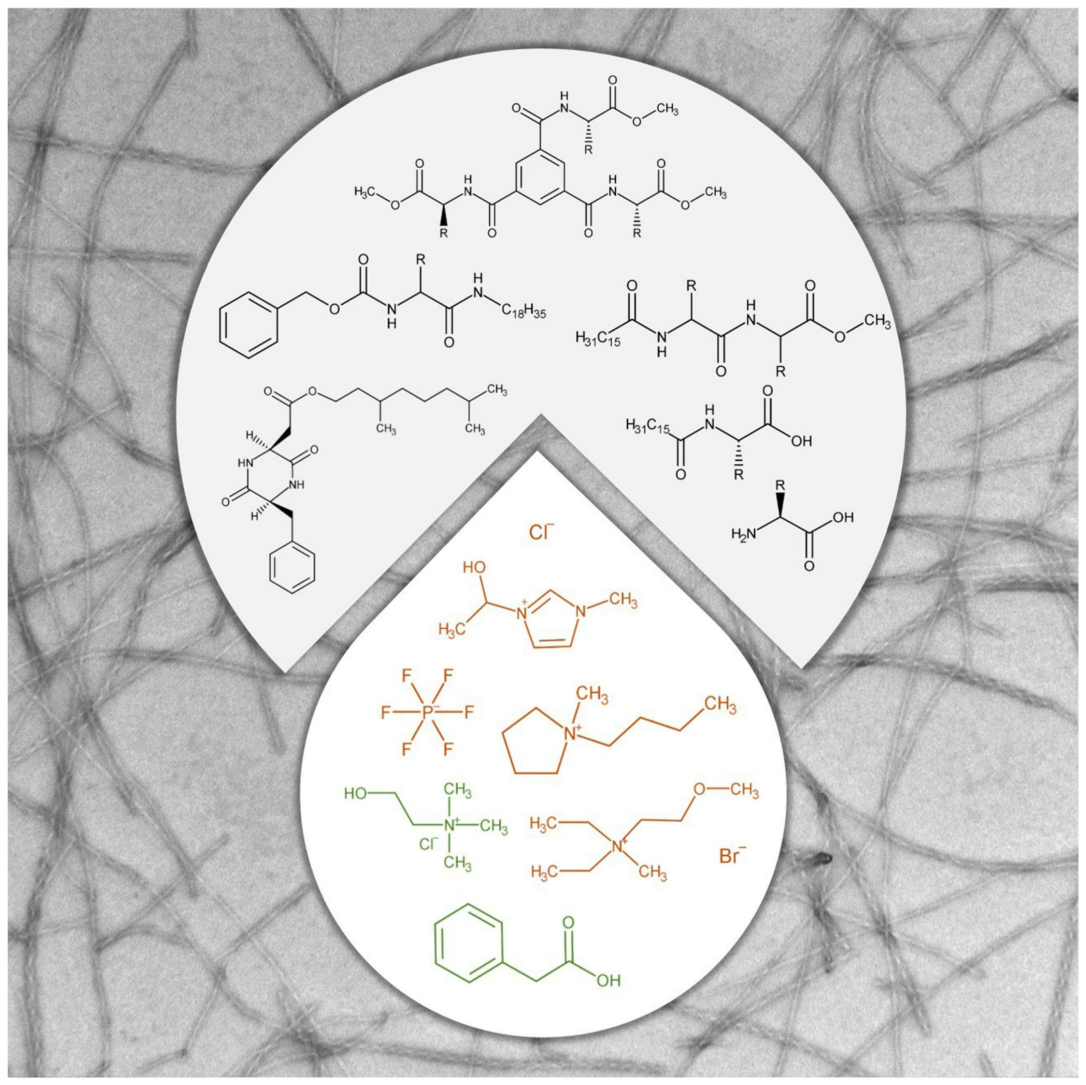

## 1 Introduction

**Scheme 1:**
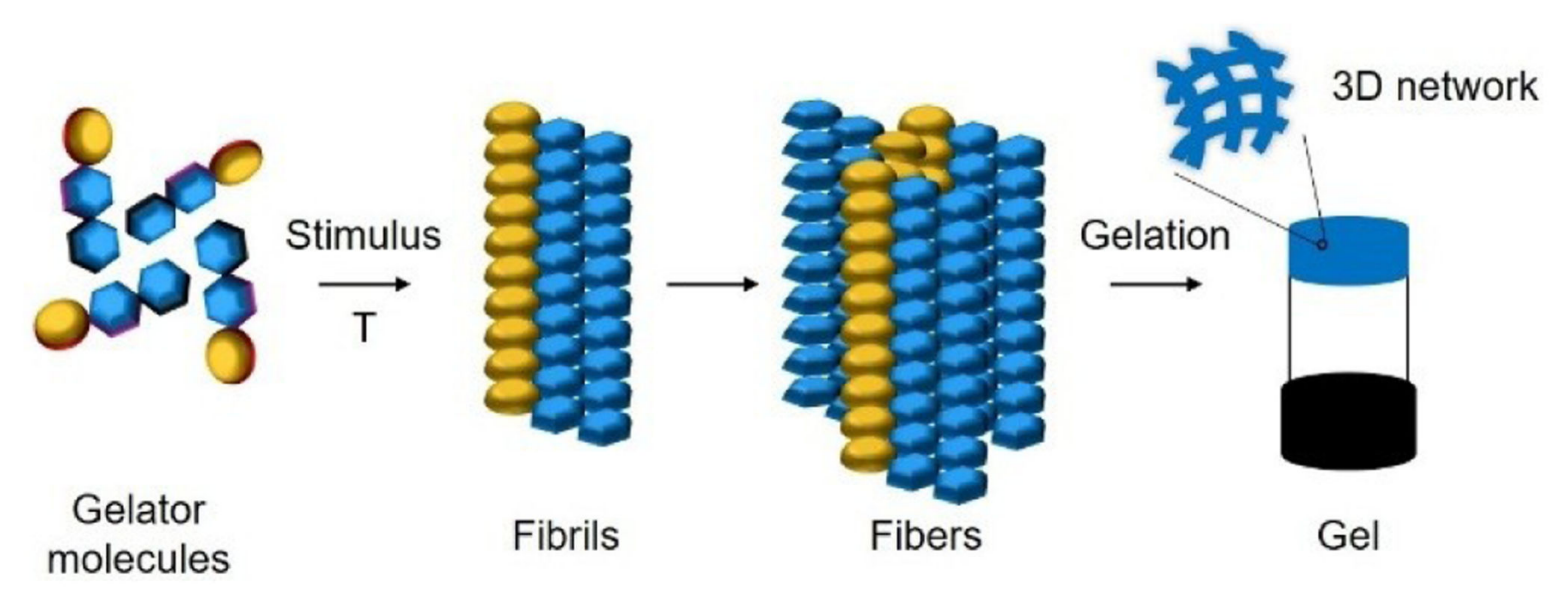
Schematic representation of supramolecular gelation.

The supramolecular self-assembly of small molecules (LMW molecules with molecular weight <2 kDa), as peptides or amino acids, in aqueous systems, leading to the formation of soft biomaterials has been widely explored in various fields over the last few decades, including bioelectronics,^[1]^ cosmetics^[2]^ and biomedicine.^[3]^ The supramolecular self-assembly process follows a bottom-up approach where simple molecules, known as the gelator molecules, undergo a stimulus (e. g. temperature change), initiating self-assembly to form fibrils. These fibrils further evolve into fibers, ultimately resulting in the development of 3D network that entraps the solvent, giving rise to a gel (Scheme 1). The primary driving forces behind the self-assembly and gelation process are cooperative non-covalent interactions among the gelator molecules, including hydrogen bonding (H-bonding), Van der Waals forces, π-π and electrostatic interactions. Hydrogels, formed by entrapping aqueous solvents within the 3D network, exhibit a significant limitation when exposed to environmental conditions. This limitation is associated with the structural stability of the 3D network, influenced by water evaporation.

**Figure 1:**
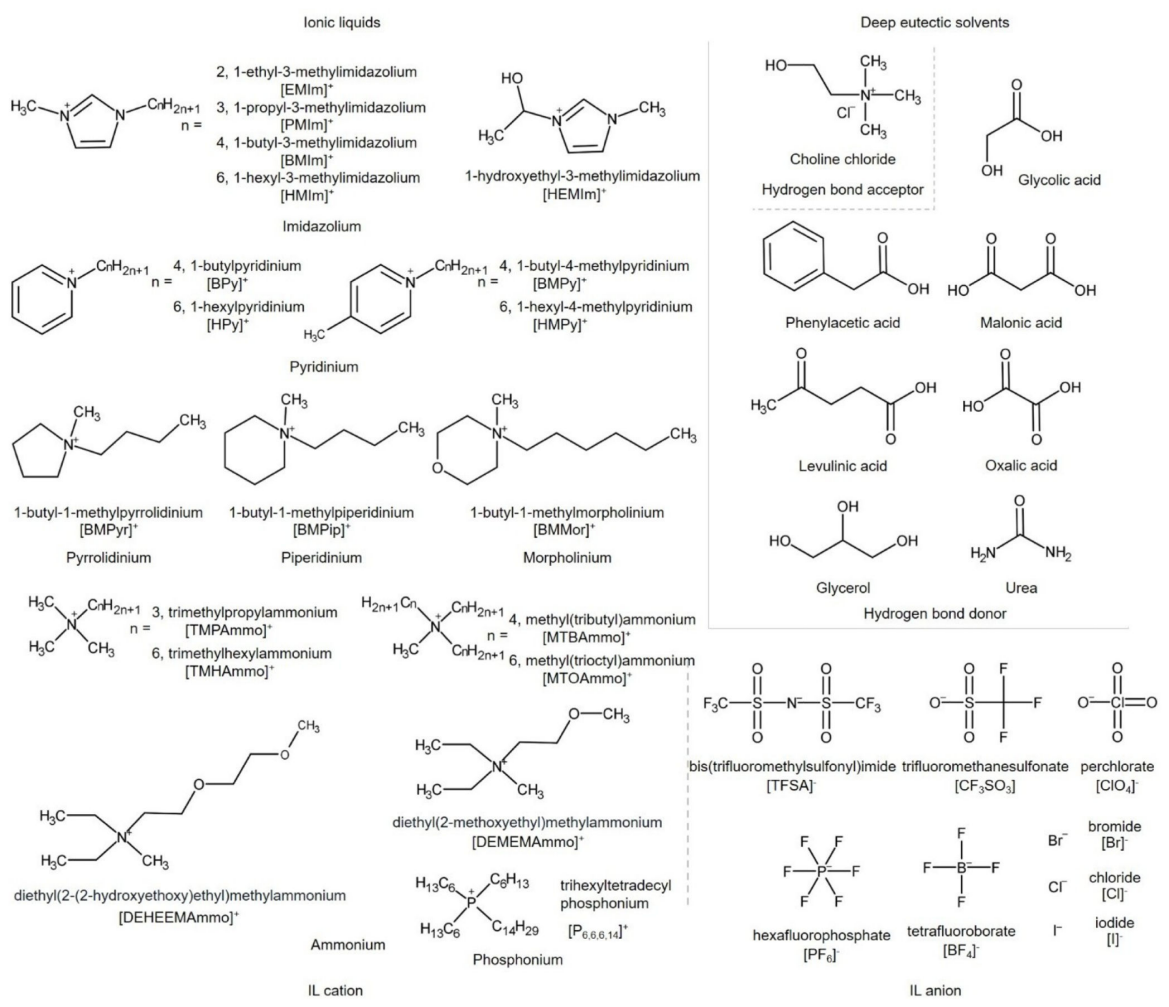
Chemical structures of ionic liquids and deep eutectic solvents found in ionogels and eutectogels.

Non-conventional solvents, such as ionic liquids (ILs) and deep eutectic solvents (DESs) overcome this limitation due to their unique characteristic of negligible vapor pressure. ILs and DESs are also known for their low flammability and high solubilization ability for biomolecules. The key distinction between these two solvents lies in the fact that ILs consist of pure components, while DESs are mixtures. ILs, classified as molten salts or salts melting bellow 100 °C, are composed of an organic cation and an organic or inorganic anion.^[4]^ Their physicochemical properties can be easily tuned through simple combinations of IL cations, with the most common belonging to imidazolium, pyridinium, piperidinium, tetraalkylammonium and tetraalkylphosphonium-based families, and IL anions such as tetrafluoroborate, hexafluorophosphate, dicyanamide, thiocyanate, bis(trifluoromethylsulfonyl)imide or trifluoromethanesulfonate (Figure 1). On the other hand, DESs are a mixture of two or more compounds with a melting point lower than its individual components.^[5]^ The most common type of DESs (type III) comprises mixtures of an organic salt (hydrogen bond acceptor, HBA) such as quaternary ammonium salts and a molecule compound (hydrogen bond donors, HBD) as amides, carboxylic acids or carbohydrates.

A gelator molecule can be classified based on its ability to self-assemble in a specific solvent. A molecule capable of self-assembly within an IL, resulting in an ionogel, is referred to an ionogelator. Conversely, a molecule able to self-assembly in a DES, forming an eutectogel, is an eutectogelator.

Over the last 23 years, LMW molecules have been reported as ionogelators and/or eutectogelators including peptide amphiphile molecules,^[6,7]^ amino acid,^[8–10]^ amino acid protected^[9,11]^ or amino acid-based amphiphile molecules,^[7,12–20]^ sugar-based gelators,^[21–24]^ carbohydrates,^[25]^ cholesterol-based,^[15,26]^ and metalogelators.^[27,28]^

**Figure 2:**
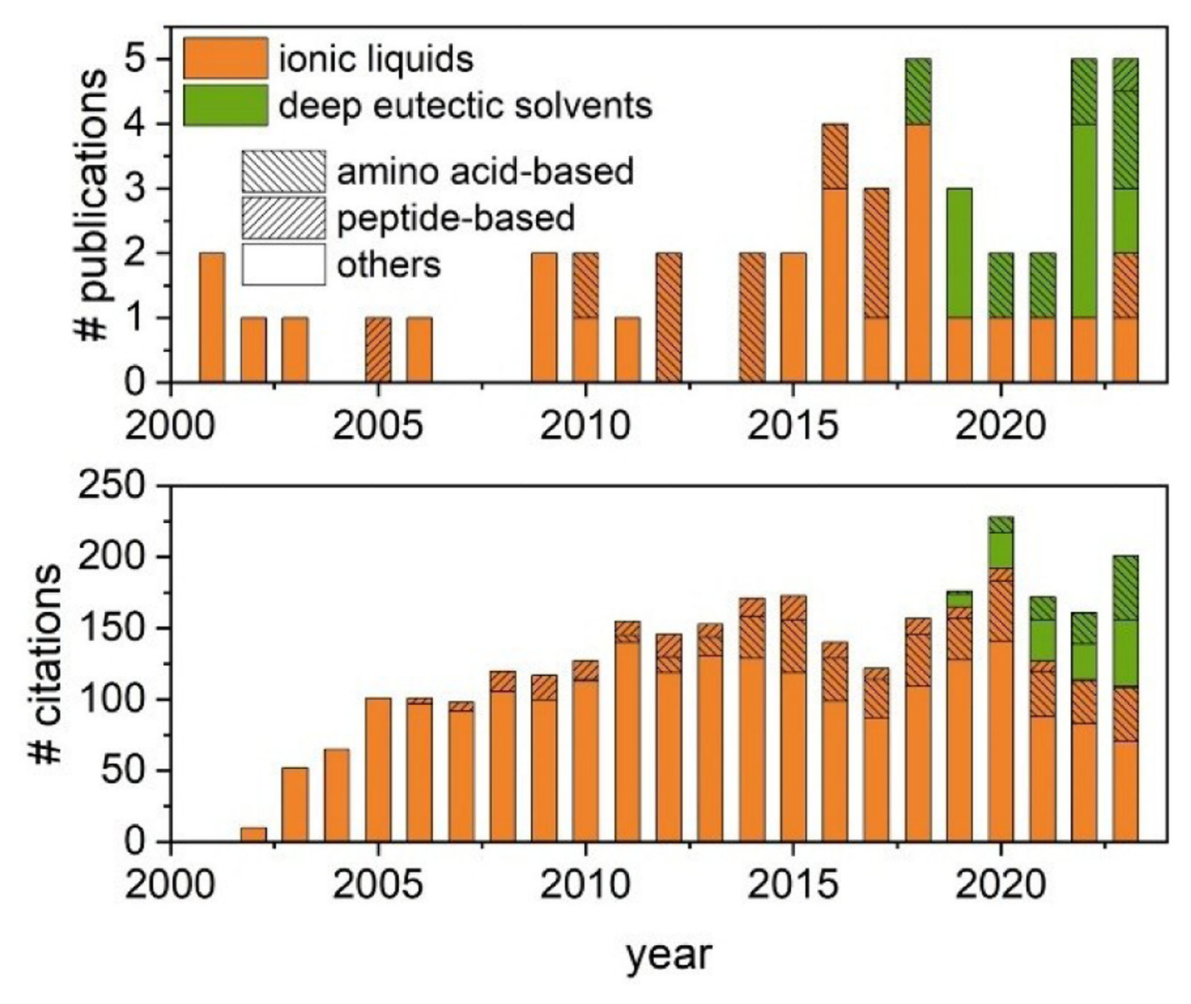
Number of publications and citations on low molecular weight gelators in the non-conventional solvents, ionic liquids and deep eutectic solvents, in the period 2001-2023 (Scopus, accessed on January 2024).

The first example of a LMW ionogelator was reported by Kimizuka and Nakashima in 2001, where a synthetic glycolipid molecular self-assembled to form a glycolipid bilayer membrane and an ionogel.^[25]^ Hanabusa *et al*. have published the first report on peptide ionogelators^[6]^ a few years later. The concept of amino acid-based amphiphile ionogelator was introduced in 2010 by Dutta and co-workers.^[13]^ The emergence of DESs in the field of biomaterials led to the first LMW eutectogelator: Marullo *et al*. reported on the simplest biological building block in aqueous solvent, an amino acid, demonstrating its ability to developed a 3D network in DESs giving rise to a supramolecular eutectogel.^[8]^ Since then, the number of publications on LMW gelators in non-conventional solvents indicates a shift in the development of these soft supramolecular materials towards DESs-based over ILs-based materials (Figure 2). This shift might be associated with DESs (discussed) greener nature, low cost, easy production process and no waste generation. In recent years, researchers have highlighted and analyzed the progress and strategies in the development of novel iono- and eutectogelator molecules, contributing to the generation of new soft ionic materials,^[4,29,30]^ particularly in the field of bioelectronics. Special emphasis has been given to LMW ionogelators^[31]^ and eutectogelators,^[32]^ where reports on supramolecular self-assembly remain limited (Figure 2), and associated with serendipity. To the best of our knowledge, there is no report associated with the rational designed of LMW supramolecular gels in non-conventional solvents, ILs or DESs. Previous publications have discussed structure-property correlation between small molecules and their self-assembly ability in ILs^[13]^ and DESs,^[7]^ emphasizing the crucial need of a hydrophiliclipophilic (or hydrophobic) balance, as seen in other supramolecular systems.^[33]^

In this work, our emphasis is on the serendipitous process of supramolecular self-assembly in ILs and DESs, leading to the formation of ionogels and eutectogels. Our focus is on systems utilizing unmodified or modified amino acids and peptides as supramolecular gelators. Furthermore, we present in-depth insights into potential strategies and approaches employed in ionogelation and eutectogelation processes as reported so far. Overall, our goal is to provide a comprehensive and up-to-date review, serving as a key bibliographic source for aspiring and experienced researchers.

## 2 Peptides as Iono- and Eutectogelators

Peptides encapsulate both the functional and structural characteristics inherent in proteins. Comprising amino acids, peptides simplify the complexity of proteins, providing a rational approach for the design of innovative and unique functional soft materials. Extensive research has been conducted on the potential self-assembly peptides to generate functional nanostructures in aqueous systems.^[34]^ However, the exploitation of peptide self-assembly in non-conventional solvents is still in its infancy, as reflected in Table 1. To our knowledge, only short peptides or short peptide-based molecules were able to form a gel in ILs and DESs. The (cyclo)dipeptides, cyclo(L-β-3,7-dimethyloctylasparaginyl-L-phenylalanyl) and cyclo(L-β-2-ethyl-hexylasparaginyl-L-phenylalanyl), were described as ionogelators^[6]^ while dipeptide-based amphiphiles were shown to be eutectogelators.^[7]^

**Figure 3:**
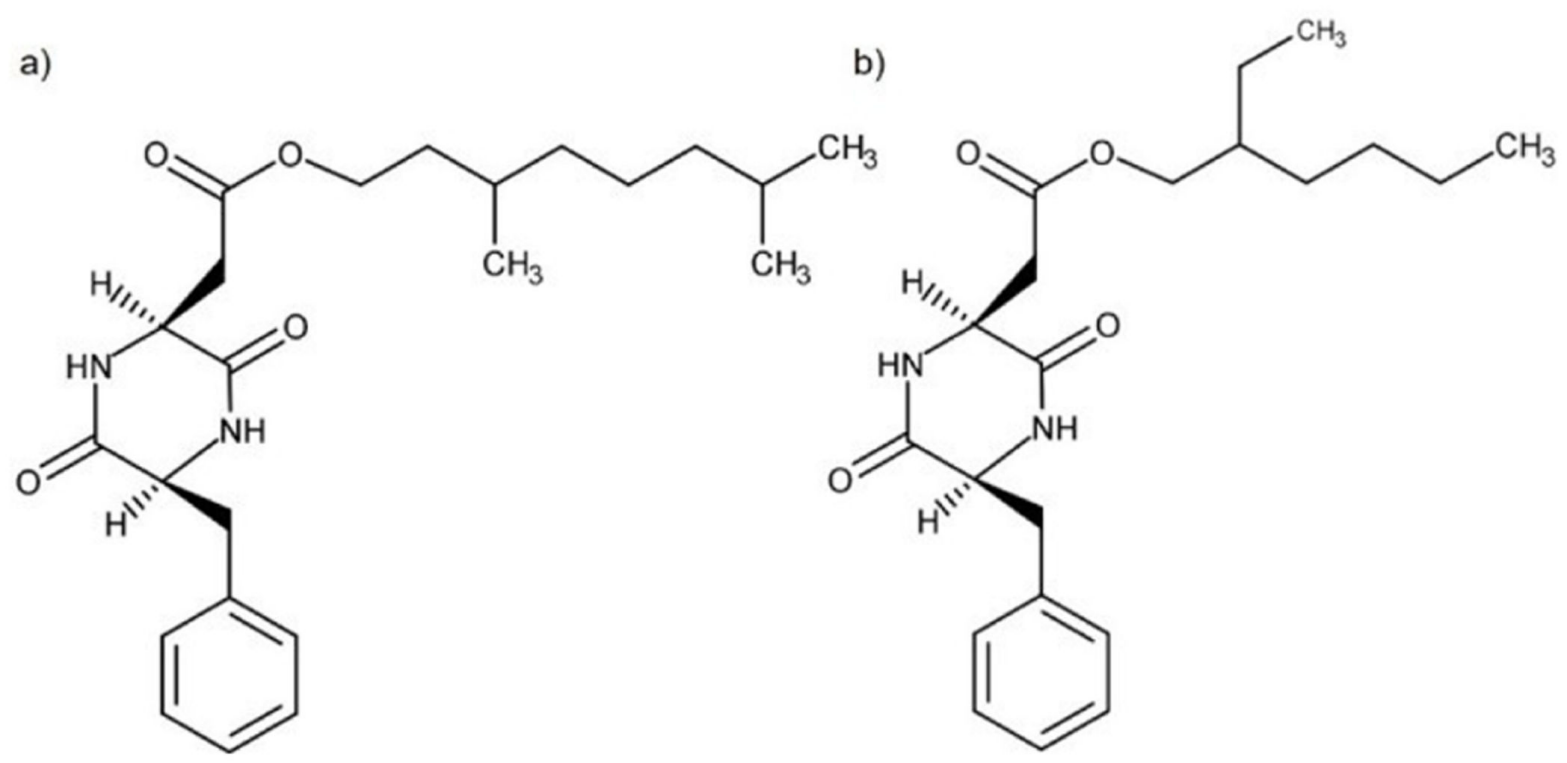
Chemical structures of a) cyclo(L-β-3,7-dimethyloctylasparaginyl-L-phenylalanyl) and b) cyclo(L-β-2-ethylhexylasparaginyl-L-phenylalanyl).

Cyclo(dipeptides) cyclo(L-β-3,7-dimethyloctylasparaginyl-L-phenylalanyl) and cyclo(L-β-2-ethylhexylasparaginyl-L-phenylalanyl) (Figure 3) where the first and to our knowledge, the only peptide ionogelators.^[6]^ These gelator molecules can form highly intertwined fibers, leading to the gelation of a wide variety of ILs (Table 1) at a minimum gelation concentration (MGC) below 1.5%w/v. The exception was observed for 1-butyl-3-methylimidazolium trifluoromethanesulfonate ([BMIm][CF_3_SO_3_]), 1-hexyl-3-methylimidazolium iodide ([HMIm][I]) and diethyl(2-(2-hydroxyethoxy)ethyl)methylammonium bis(trifluoromethylsulfonyl)imide ([DEHEEMAmmo][TFSA]) with MGCs of 2 %w/v or higher. However, Hanabusa and co-workers have disregarded the influence of IL nature in the gelation process, emphasizing the importance of the gelator molecule’s chemical structure and its contribution for supramolecular self-assembling, particularly in creating the H-bonding network and Van der Waals interactions between the fibers. The presence of a branched alkyl group (3,7-dimethyloctyl or 2-ethylhexyl) onto the cyclo(dipeptide) core was crucial for the ionogelation process occur, as its substitution by long natural alkyl groups as C_18_H_37_ or C_11_H_23_ led to no gel formation. Considering the two gelator molecules within this study, cyclo(L-β-3,7-dimethyloctylasparaginyl-L-phenylalanyl) exhibited higher gelation ability (i. e. lower gelator MGCs) than cyclo(L-β-2-ethylhexyl asparagin-yl-L-phenylalanyl).

In recent years, studies on the potential self-assembly of unprotected dipeptides in ILs have been reported, although the successful formation of an ionogel was not achieved. The unprotected dipeptide L-Phenylalanine-L-Phenylalanine (L-Phe-L-Phe) demonstrated the ability to form nanostructures in both hydrophilic (1-butyl-3-methylimidazolium tetrafluoroborate, [BMIm][BF_4_]) and hydrophobic (1-ethyl-3-methylimidazolium bis(trifluoromethylsulfonyl)imide, [BMIm][TSFA]) ILs.^[35]^ In this study, the authors used ILs to investigate the role of water in the self-assembly process of Phe-Phe into nanotubes. The self-assembly of diimidazolium-based organic salt molecules, where the anion was the unprotected dipeptide D-Alanine-D-Alanine (D-Ala-D-Ala), in the IL 1-butyl-3-methylimidazolium hexafluorophosphate [BMIm][PF_6_] and [BMIm][BF_4_] was reported as unsuccessful by Rizzo and co-workers.^[18]^

**Figure 4:**
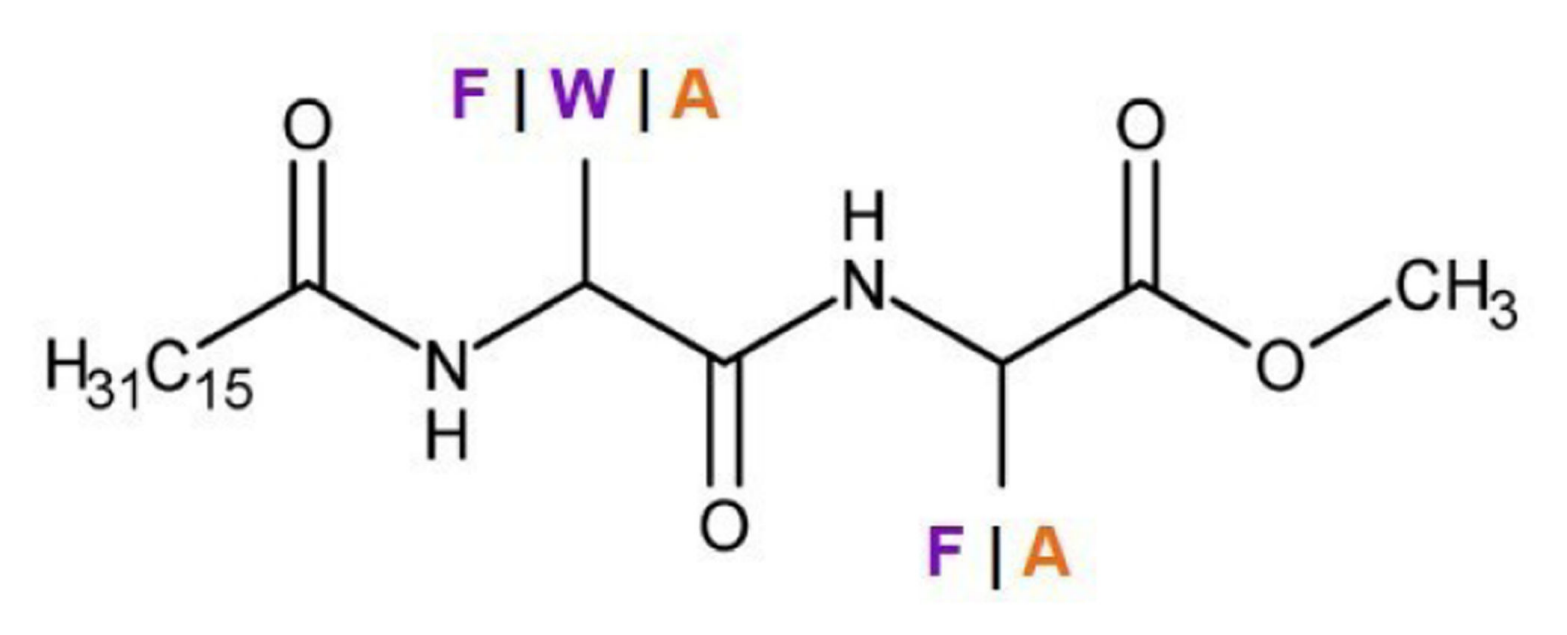
Chemical structure of peptide amphiphile eutectogelators. F: phenylalanyne. W: tryptophan. A: alanine. Amino acid: Aliphatic amino acid. Aromatic amino acid.

Recently, Khan and co-workers reported the first peptide amphiphile eutectogelators. They explored the use of dipeptide amphiphile-based molecules, incorporating the aromatic amino acids, L-tryptophan (L-Trp) and L-Phe, as well as the aliphatic amino acid L-Ala, to act as gelator molecules in the hydrophilic DES, choline chloride:phenylacetic acid with a molar ratio of 1 : 2 (Table 1, Figure 1).^[7]^ In this study, the peptide amphiphile molecules consisted on a dipeptide as the central unit, modified at the N-terminus with a palmitic acid’s C-16 hydrophobic chain and at the C-terminus with a methyl ester (Figure 4). The Phe-Phe-based amphiphile gelator demonstrated excellent gelation ability in choline chloride:phenylacetic acid with a MGC of 1.0%w/v. The substitution of one L-Phe with the also aromatic residue L-Trp, at the hydrophobic end of the C-16 alkyl tail (Trp-Phe-based amphiphile molecule) led to a decrease on the gelation ability, with an increased MGC of 10.0 %w/v. This observation highlighted the importance of the nature of the amino acid side chain in the gelation process. The change of one hydrophobic amino acid, L-Phe, by an amino acid with hydrophilic character, L-Trp, modified the hydrophilic-lipophilic character of the amphiphile gelator and consequently, its gelation ability towards the solvent in study. The importance of this balance was strengthen with the substitution of both L-Phe residues in the amphiphile molecule by L-Trp residues. The Trp-Trp-based amphiphile molecule has a more hydrophilic character than the previous dipeptide amphiphile eutectogelators, and was unable to form a gel in the DES choline chloride: phenylacetic acid. The dipeptide amphiphile with the aliphatic hydrophobic amino acid L-Ala (Ala − Ala-based amphiphile molecule) demonstrated moderate gelation efficiency in the aromatic ring-containing DES with a MGC of 5.0%w/v.

## 3 Amino Acid-Based Iono- and Eutectogelators

**Figure 5:**
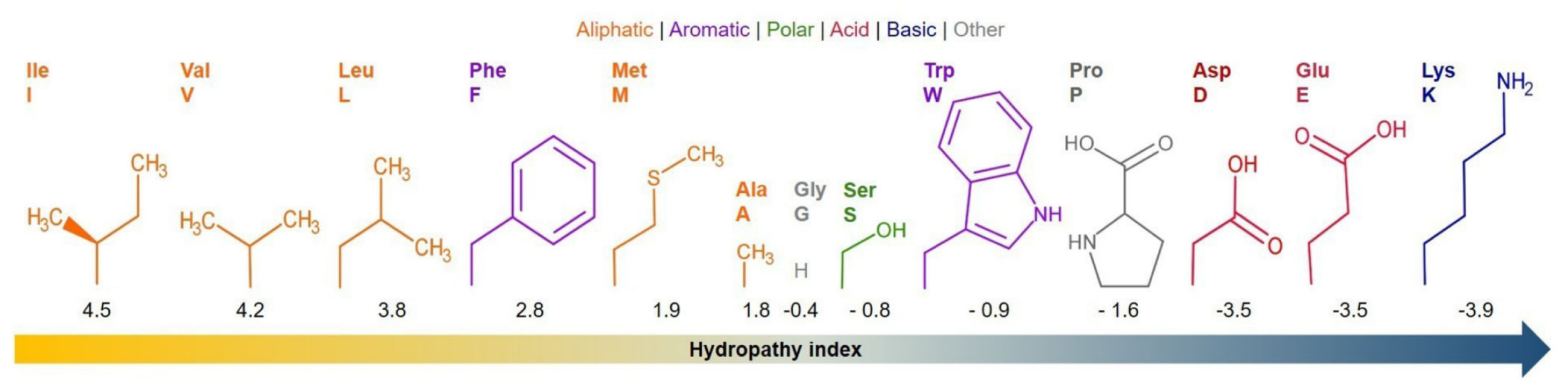
Hydropathy index and chemical structures of amino acid side chains found in ionogelators and eutectogelators.

Amino acids are ideal bulding blocks for soft materials owing to their versatile chemical structures and unique functional properties.^[36]^ With chemically diverse side chains, they allow for the tailored design of biomaterials with specific functionalities. Their remarkable self-assembly ability, driven by non-covalent interactions, facilitates the creation of biomaterials with well-defined structures. The hydropathy index,^[37]^ a measure commonly used to quantify the hydrophobicity or hydrophilicity of amino acids, plays a crucial role in understanding their interactions in biological and biomimetic systems. This index assigns numerical values to amino acids based on their affinity for water (Figure 5), guiding the design of biomaterials and the study of self-assembly processes. While initially applied to proteins, the hydropathy index can potentially be extended to evaluate the behavior of amino acids in ILs^[13]^ and DESs.^[7]^ In these non-conventional solvents, the hydropathy index may offer insights into the interactions between amino acids and the solvent environment, influencing the design and understanding of soft materials in these unique settings.

Amino acid self-assembly in non-conventional solvents will be discussed based on the chemical modification of the amino acid molecule: unmodified amino acid (Table 2), protected amino acid (Table 3), modified amino acid-based amphiphiles (Table 4), and other amino acid-based (Table 5) molecules. One curiosity concerns the fact that, at the moment of writing this review, the gelation ability of unmodified amino acid and protected amino acid molecules in non-conventional solvents has been reported only for DESs; there was no evidence of being ionogelators. The amino acid-based eutectogels were developed using DESs where choline chloride was the HBA specie, while the HBD were acids (phenylacetic acid, leuvonic acid, malonic acid, oxalic acid, glycolic acid), glycerol and urea. Amino acid-based ionogels were developed in hydrophilic ILs, where IL cation predominantly belong to the imidazolium-based family and IL anion were fluorinated ions, bis(trifluoromethylsulfonyl)imide ([TFSA]^−^), tetrafluoroborate ([BF_4_]^−^), hexafluorophosphate ([PF_6_]^−^) or trifluoromethanesulfonate ([CF_3_SO_3_]^−^). IL cations from other families such as piperidium, pyrolidinium, phosphonium, morpholinium or ammonium were also studied, as well as IL anions, chloride ([Cl]^−^) or bromide ([Br]^−^).

### 3.1 Amino Acids

**Figure 6:**
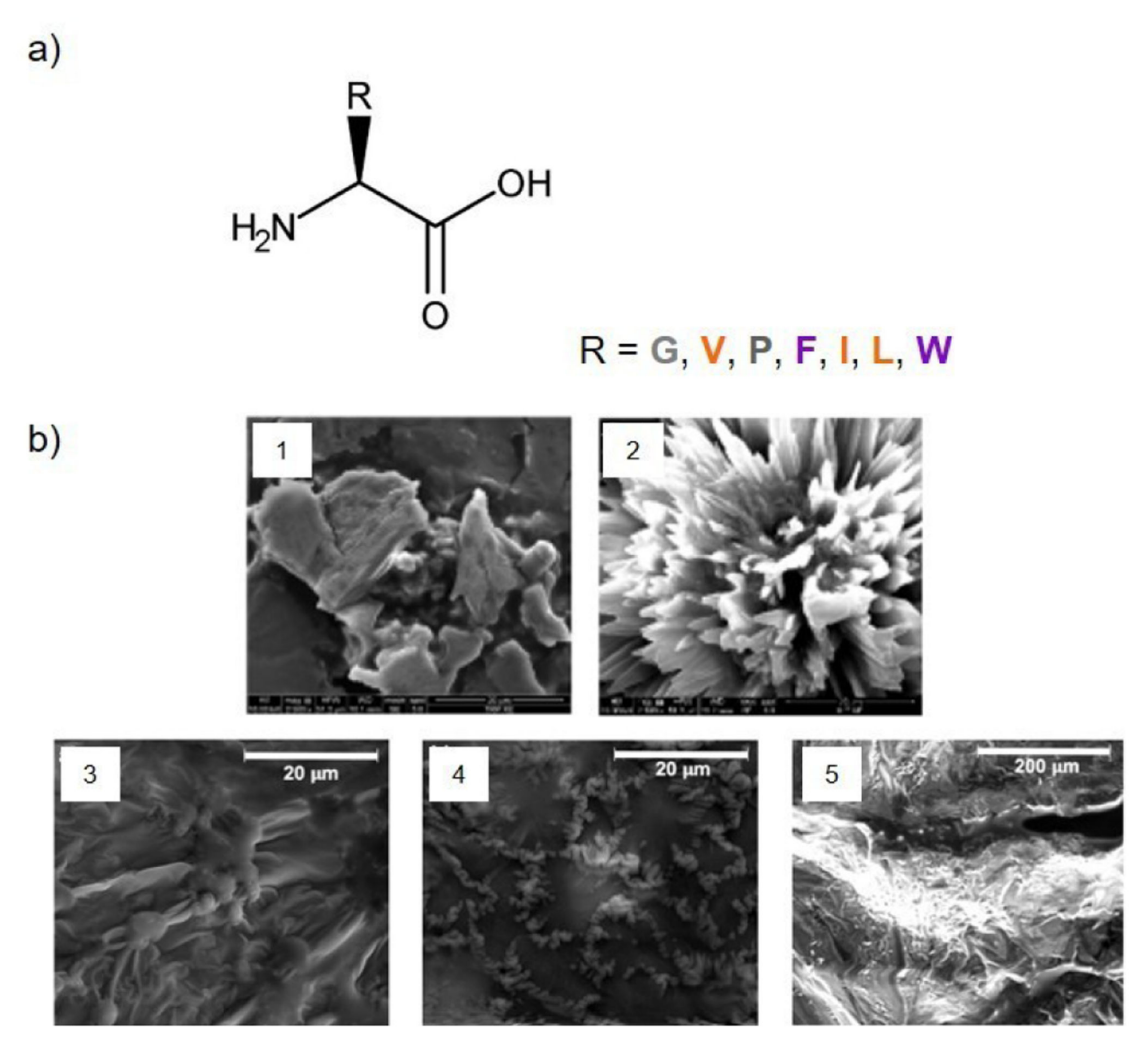
Amino acid eutectogelators. a) Chemical structure. b) SEM images of euctectogels in choline chloride:phenylacetic acid (1 : 2) at 3 wt% of gelator formed by 1) L-Tryptophan, 2) L-Isoleucine, Adapted with permission from ref.^[8]^ Copyright 2018. American Chemical Society; 3) L-Leucine, 4) L-Proline, and 5) L-Phenylalanine. Adapted with permission from ref.^[9]^ Copyright 2020. John Wiley and Sons. G: glycine. V: valine. I: isoleucine. L: leucine. F: phenylalanine. P: proline. W: tryptophan. Amino acid: Aliphatic amino acid. Aromatic amino acid. Other amino acid.

Self-assembly of unmodified amino acids (Figure 6a) in the DESs choline chloride:phenylacetic acid (1 : 2) and/or choline chloride: urea (1 : 2) was reported for the aromatic amino acids Trp and Phe, the aliphatic amino acids isoleucine (Ile) and leucine (Leu), the polar amino acid serine (Ser) and the amino acid proline (Pro)^[8–10]^ (Table 2).

The gelation ability of simple biological molecules, such as the aromatic amino acid residue L-Trp, and the aliphatic amino acid residue L-Ile, in the hydrophilic aromatic-containing DES choline chloride:phenylacetic acid (1 : 2) (Figure 1) was first explored by Marullo and co-workers.^[8]^ These two amino acids were the first examples of LMW eutectogelators, producing opaque eutectogels at a gelator concentration of 2-5 wt%. The gelation of the DES was faster for L-Ile as gelator molecule, resulting in stronger eutectogels compared to L-Trp, whose emissive properties (showed by fluorescence) were inherited by the developed eutectogel. The different morphologies, wind-flower-like structures for L-Ile eutectogel and flake-like structures for L-Trp eutectogel (Figure 6b), arose from the interactions in the gel network, where the nature of the amino acid side chain seemed to play a crucial role. Later, the same authors extended their study to other amino acids residues as the aromatic L-Phe, the aliphatic, L-Leu or the amino acid L-Pro.^[9]^ In this work, it was not only tested the aromatic-containing DES choline chloride:phenylacetic acid (1 : 2) but also the non-aromatic DESs choline chloride:glycerol (1 : 2) and choline chloride:ethylene glycol (1 : 2). Nonetheless, unprotected amino acid residues used in this study were only able to form an opaque gel at gelator concentration of 2-5 wt% (2-3 wt% for the aromatic amino acid L-Phe) in the aromatic-containing DES, choline chloride:phenylacetic acid (1 : 2), suggesting a strong influence of DES HBD component in the self-assembly process. As previously observed,^[8]^ the amino acid side chain play a role in the morphology of self-assembly nanostructures: L-Phe-based gel showed a very thick appearance without any particular features, while L-Pro-based gel was characterized by uniformly sparse spherical objects and L-Leu-based gel by intertwined fibers connected by spheroidal structures (Figure 6b).

L-Trp and L-Pro also self-assembled in the DES choline chloride:urea (1 : 2), forming opaque gels,^[10]^ as in the aromatic containing DES choline chloride:phenylacetic acid (1 : 2).^[8,9]^ Nonetheless, there seemed to be an increase in the gelation ability for L-Pro when DES HBD possessed aromatic and acidic chemical groups as the gelator concentration needed to form a stable eutectogel was lower (2 wt% *vs* 3 wt%). L-Trp was able to form a gel in both DESs at a gelator concentration of 2 wt%.

The aliphatic hydroxyl group-containing amino acid, L-serine (L-Ser), was also able to self-assemble in the DES choline chloride:urea (1 : 2) forming opaque eutectogel at gelator concentration 3.0 wt%,^[10]^ as discussed previously for amino acid. Additionally, Saavedra and co-workers unsuccessfully explored the eutectogelation ability of the aliphatic amino acid, L-Ile, the positively charged amino acid L-asparagine (L-Asp) and the amino acid L-cysteine (L-Cys). Furthermore, the amino acid L-Pro was modified to raise its derivatives L-prolinamide (L-Pro-NH_2_) and *trans*-4-hydroxy-L-proline (t-4-OH−L-Pro). The success-ful eutectogelation of these molecules for a gelator concentration of 3.0 wt% showed an increase in their eutectogelation ability, emphasizing the importance of the functionalization of both the carboxyl group and aliphatic skeleton of the L-Pro.

### 3.2 Protected Amino Acids

**Figure 7:**
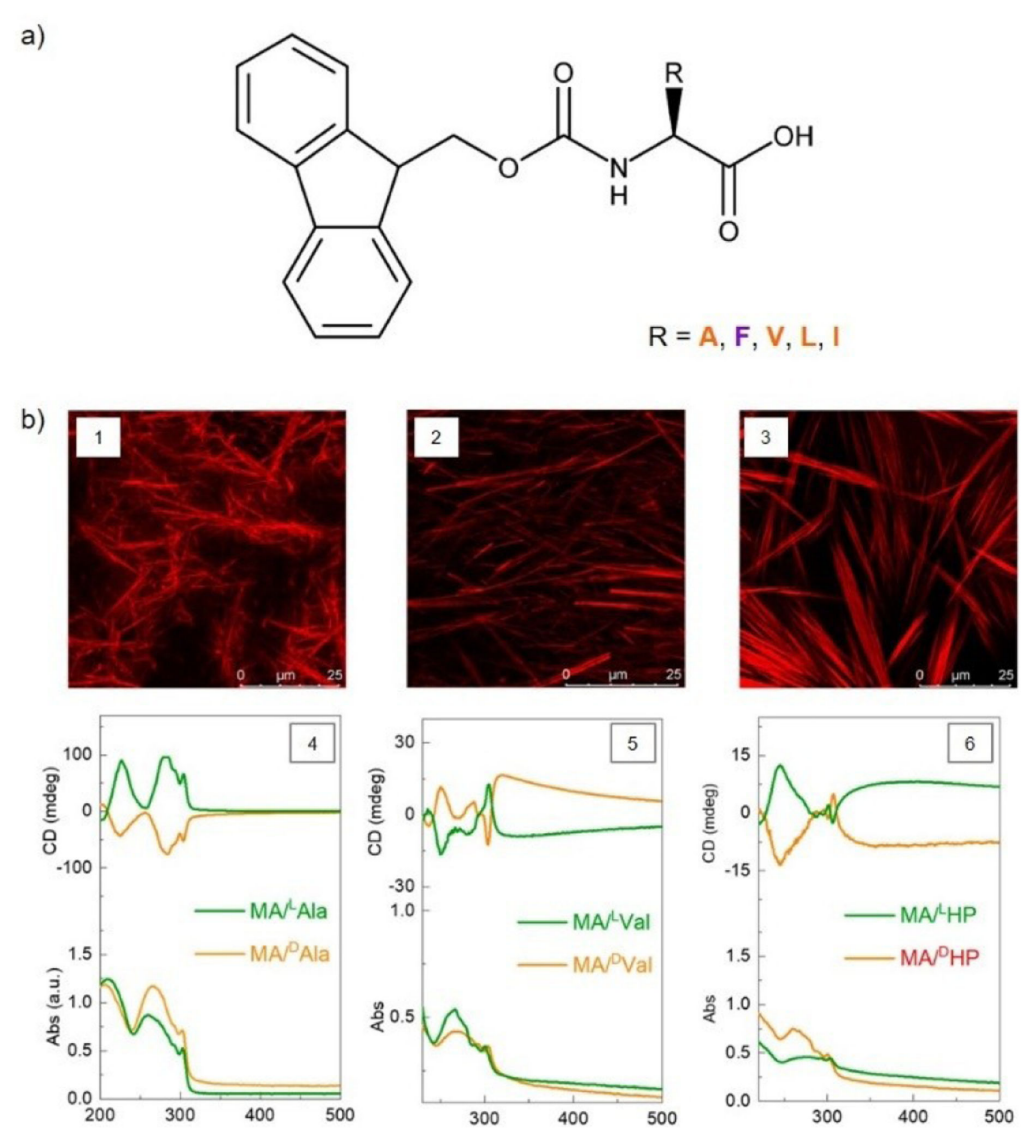
a) Chemical structure of Fmoc-protected amino acid eutectogelators. A: alanine. F: phenylalanine. V: valine. L: leucine. I: isoleucine. Aliphatic amino acid. Aromatic amino acid. b) Fmoc protected amino acid eutectogelators: Confocal microscopy images of self-assembly nanostructures in DES choline chloride:malonic acid 1) Fmoc-L-Ala, 2) Fmoc-L-Val, 3) Fmoc-L-Homophenylalanine and corresponding circular dichroism spectra (4), 5) and 6)). Adapted with permission from ref.^[11]^ Copyright 2022 American Chemical Society.

Amino acids residues protected at the N-terminus with the bulky aromatic group 9-fluorenylmethoxycarbonyl (Fmoc) group (Figure 7a) self-assembled in DES choline:phenylacetic acid (1 : 2),^[9]^ choline chloride:malonic acid (2 : 2), choline chloride:oxalic acid (2 : 2) and choline chloride:glycerol (2 : 4)^[11]^ (Table 3).

Fmoc protected aromatic and aliphatic amino acid residues, Fmoc-Phe and Fmoc-L-Ile, produced opaque eutectogels at a gelator concentration 2-5 wt% in the aromatic containing DES choline chloride:phenylacetic acid (1 : 2).^[9]^ In comparison with their respective unmodified amino acids, the presence of a Fmoc group at the N-terminus of the amino acid led to antagonist effects based on amino acid side chain: for the aliphatic amino acid L-Ile, functionalizing the N-terminus with a Fmoc group increased eutectogel thermo stability, while in the aromatic residue L-Phe, there was no significant change. The change in gelation ability for Fmoc-L-Ile over L-Ile was associated with the existence of aromatic and aliphatic moieties in the eutectogelator molecule. Similarly, nanostructures formed presented different morphologies: Fmoc-L-Ile were uniformly sparse spherical objects, whereas Fmoc-L-Phe fomed a fiber network coiled into honey comb shaped structures. In the DES choline chloride:malonic acid (2 : 2), 7% mol (MGC) Fmoc-Phe self-assembled into a 3D gel network with condensed fibrous structure, as did Fmoc-HP (MGC 5 mol%), Fmoc-Leu (MGC 3 mol%), Fmoc-Val (MGC 1 mol%) and Fmoc-Ala (MGC 7 mol%).^[11]^ Morphological characterization showed no helical-like structures; nevertheless, supramolecular chirality was demonstrated by circular dichroism for all developed Fmoc-amino acid-based gels in choline chloride:malonic acid (Figure 7b). Supramolecular chirality pertains to the asymmetrical organization of the molecules with self-assembly ability, as oppose to molecular chirality determined by molecular configuration. The influence of the solvent on the self-assembly process was previously shown in other systems. Fmoc-Ala was also able to self-assembled in DES choline chloride:oxalic acid (2 : 2) and choline chloride:glycerol (2 : 4).

Molecular dynamics studies were employed to provide insights into self-assembly behaviors in DES. The results suggest that the aggregation of Fmoc-amino acids can be triggered in the presence of the DES systems, and the carboxylic groups of the building units were actively involving with DES.

The eutectogelation ability of other Fmoc-protected amino acid was also explored. Fmoc-Asp self-assembly in choline chloride:malonic acid (2 : 2) gave rise fibrous nanostructures; however, no indication was given by the authors that a eutectogel was form. Fmoc-Pro in choline chloride:malonic acid formed an emulsion, while Fmoc-Ser revealed high solubility.^[11]^

### 3.3 Amino Acid-Based Amphiphiles

**Figure 8:**
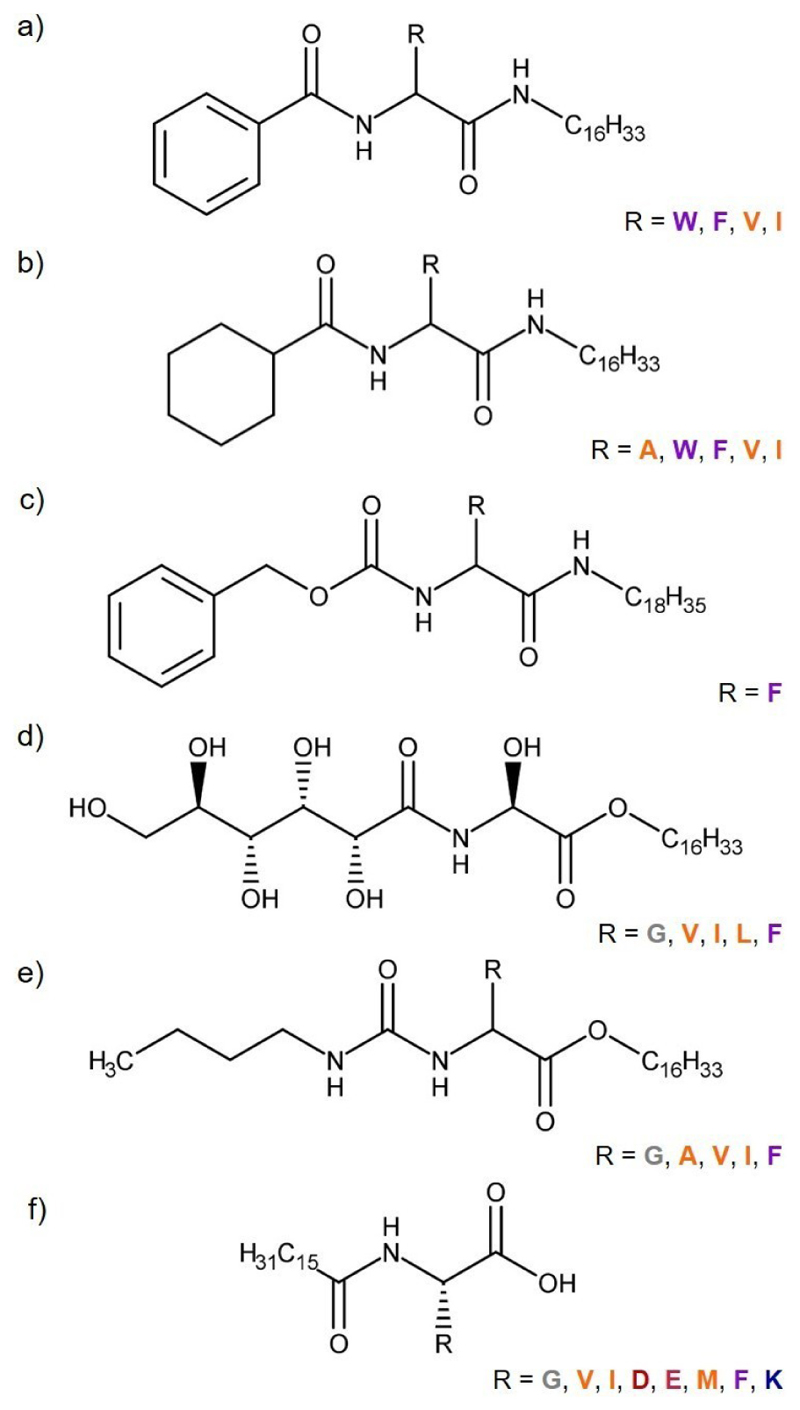
Chemical structures of amino acid-based amphilile ionogelators and eutectogelators. A: alanine. W: tryptophan. F: phenylalanine. V: valine. I: isoleucine. G: glycine. L: leucine. D: aspartic acid. E: glutamic acid. M: methionine. K: lysine. Amino acid: Aliphatic amino acid. Aromatic amino acid. Acid amino acid. Basic amino acid. Other amino acid.

Amino acid-based amphiphiles are LMW molecules where the amino acid residue is bonded to one or more aliphatic tails. The amino acid residue is the central element of the amphiphilic molecule, modified at N-terminus, C-terminus or both. Amino acid-based amphiphile molecules have shown ability to form gels both in ILs and DESs (Table 4). Interestingly, the majority of the amino acid-based ionogelators reported so far are amino acid-based amphiphiles^[13–15,17,20]^ (Figure 8) with different gelation ability towards hydrophilic ILs, where IL cation was predominantly from the imidazolium-based family and IL anion was a fluorinated ion, [TFSA]^−^, [BF_4_]^−^, [PF_6_]^−^ or [CF_3_SO_3_]^−^. IL cations from other families as piperidium, pyrolidinium, phosphonium, morpholinium or ammonium were also studied as well as IL anions, chloride ([Cl]^−^) or bromide ([Br]^−^). Amino acid-based amphiphile eutectogelators reported the gelation of DESs where the HBA was choline chloride combined with acid compounds (phenylacetic acid, levulinic acid, glycolic acid) or glycerol as HBD.^[7,12]^

Overall, all amino acid-based amphiphile ionogelators and eutectogelators are chemically modified at both termini, N-terminus and C-terminus. In these gelator molecules, the long aliphatic chain is at the C-terminus.^[7,13,14,17,20]^ The only exception is for an amino acid-based ionogelator molecule reported by Restu *et al*. where the amino acid is only modified at the N-terminus with a long aliphatic chain.^[19]^ (Figure 8). Small changes in the chemical structure of amino acid-based amphiphile molecules, as the length of the aliphatic tail, the use of protecting groups or substitution of amino acid residue, might strongly affect its gelation ability as the its chemical nature and, in particular its hydrophilic-lipophilic character, is altered.^[7,13]^

The ionogelation ability of amino acid-based amphiphile molecules within the ILs from 1-butyl-3-methylimidazolium and 1-butylpyridinium families, in the absence (hydrophilic ILs) or presence of 10 % water (solid ILs), was initially investigated by Dutta and co-workers.^[13]^ The most effective ionogelator molecules emerge when amino acid-based amphiphile molecule underwent modifications at both termini: a long C16 hydrophobic chain at the C-terminus and either a phenyl ring or a non-planar cyclohexyl group (more hydrophobic moiety) at the N-terminus (Figures 8a and 8b). The study considered the aromatic amino acid residues, L-Trp and L-Phe, and the aliphatic residues, L-Ile, L-Val and L-Ala, revealing different gelation abilities towards the ILs. The aromatic amino acid residues, L-Trp and L-Phe, exhibit best gelation ability when the N-terminus was modified with the cyclohexyl group. This modification enhanced intermolecular H-bonding by aligning perpendicular to the amide groups, enabling the formation of a gel with comparable gelator MGC (1.3-2.1 %w/v) in all the tested ILs for L-Trp amphiphile molecule. Despite cyclohexyl-modified L-Phe amphiphile was also able to form a gel in all tested ILs, MGCs were higher (MCG 1.5-5.1 %w/v) than for L-Trp amphiphile molecule. An exception was observed for the IL [BMIm][BF_4_], where L-Phe-based amphiphile molecule demonstrated enhanced gelation ability, equal to L-Trp amphiphile ionogelator (MGC 1.5 %w/v). Moreover, phenyl-modified L-Phe-based amphiphile was able to form a gel in all ILs tested (as opposed to phenyl-L-Trp which only form an ionogel in the IL [BMIm][BF_4_]), exhibiting enhanced gelation ability (MGC 1.3-4.3 %w/v) than cyclohexyl-modified L-Phe amphiphile. This improvement was associated to the hydrophilic-lipophilic balance of the gelators, and the hydropathy index of the amino acid residues (Figure 5).

The ability to induce gelation decreased upon substituting the aromatic amino acid residue with aliphatics, L-Ile and L-Val. In contrast to the observations with the aromatic amino acid-based amphiphile molecules, the self-assembly process for these ionogelator molecules appeared to be enhanced by the presence of the phenyl group at the N-terminus, rather than the cyclohexyl group. Phenyl- and cyclohexyl-modified L-Ile amphiphiles were able to form a gel in all ILs in the study with exception of [BMIm][PF_6_]. Phenyl-modified L-Ile has shown better ionogelation ability than cyclohexyl-modified amphiphile, with MGCs of 2.5-4.6 %w/v and 3.6-4.7 %w/v, respectively. The phenyl-and cyclohexyl-modified L-Val amphiphiles have shown higher MGCs (7.8-8.2 %w/v and 7.1-10 %w/v, respectively) not being able to form a gel not only in the IL [BMIm][PF_6_] but also in the IL [BPy][BF_4_]. Notably, amphiphile molecules based on L-Ala were not able of form a gel in the studied ILs. Moreover, Dutta and co-workers emphasize the significance of the long hydrophobic chain at C-terminus. Swapping groups between termini or modifying the N-terminus with C16 alkyl chain resulted in a decrease (MGC 2.6-3.7 %w/v) or even absence of the ionogelation ability.

The amphiphile ionogelator based on L-Trp, with modification of the C-terminus with the long alkyl chain and the N-terminus with a cyclohexyl group (Figure 8b),^[13]^ exhibited also eutectogelation properties.^[7]^ It formed a gel in the hydrophilic DES choline chloride:phenylacetic acid (1 : 2) at MGC of 2.0 %w/v and choline chloride:leuvonic acid (1 : 2) at MGC of 1.0%w/v as well as the amphiphile ionogelator based on the aromatic amino acid L-Phe (MGC 1.0%w/v and 0.5%w/v, respectively) and the L-Ala-based amphiphile molecule (MGC 0.5%w/v and 1.0%w/v, respectively).^[7]^ Eutectogel formation did not occur when the N-terminus of the amino acid was modified with tertbutyloxycarbonyl (BOC) protecting group, a phenyl ring, or to the adamantane group, probably due to out-of-plane orientation. Furthermore, the impact of introducing a cyclopentyl group at the N-terminus of L-Trp-based amphiphile molecule, resulted in a reduction of gelation efficiency and increased MGC from 1.0 w/v% to 5.0 w/v%, emphasizing the crucial role of the cyclohexyl group at the N-terminus of the amino acid in eutectogelator molecules. The presence of the long C16 hydrocarbon alkyl chain at the C-terminus has also proven to be essential for the eutectogelation capability of the L-Trp-based gelator. Its substitution with the C12 hydrocarbon resulted in no gelation.

A Phe-based amphiphile molecule, wherein the aromatic amino acid residue is modified at N-terminus with a benzyloxycarbonyl group and at C-terminus with a C18 hydrophobic alkyl chain (Figure 8c), demonstrated the ability to form a gel in the hydrophobic phosphonium IL trihexyl(tetradecyl)-phosphonium bis(trifluoromethylsulfonyl)imide ([P_6,6,6,14_][TFSA]) at a MGC of 0.5 wt%. The successful formation of the gel relies in establishing the appropriate intermolecular forces between gelator with the solvent. This eutectogelator molecule exhibits a significant capability for hydrogen bonding, particularly between the N−H from the modified amino acid and O=C of the benzyloxycarbonyl group.^[20]^

Amino acid-based amphiphile molecules, modified with gluconic acid at the N-terminus and a long hydrophobic tail at the C-terminus (Figure 8d), demonstrated their gelation ability towards aqueous and organic solvents as well as ILs from imidazolium, pyridinium and piperidiumn families.^[14]^ Amphiphile molecules, with the aliphatic amino acid residues L-Val, L-Leu, and L-Ile, exhibited high ionogelation ability (MGC 0.1-1.0 wt%) in the ILs studied, compared to L-Gly (MGC 1.0-1.7 wt %) or the aromatic amino acid L-Phe (MGC 1.0-2.0 wt%). The gelation ability of amphiphile molecules was significantly reduced by the simple substitution of L-Val with D-Val with the formation of a gel at MGC of 1.0 wt% in only one of the studied ILs, [BMIm][CF_3_SO_3_], highlighting the influence of molecular chirality on gel formation. None of the tested amphiphile gelator molecules showed gelation ability towards the IL propyl(trimethyl)ammonium bis(trifluoromethylsulfonyl)imide ([TMPAmmo][TFSA]). Supramolecular ionogelators based on amino acid-derived urea were synthesized by Yu and co-workers (Figure 8e).^[17]^ These amphiphile gelator molecules were produced from hexadecyl amino acid esters (Gly, L-Ala, L-Val, L-Ile, L-Phe) and urea. The anticipated intermolecular hydrogen bonding between the amide groups and urea was thought to contributed to supramolecular self-assembly in imidazolium-based ILs. As expected, all the amphiphile molecules exhibited ionogelation ability. In particular, Gly-based amphiphile molecule demonstrated the lowest critical gelation concentration, 0.6-0.9, when compared to other amino acid amphiphile molecules in this study, which might be due to relatively low space steric hindrance. Furthermore, the impact of IL cation alkyl chain in the ionogelation ability was shown, observing a slight decrease in MGC of Gly-based amphiphile molecule with the increase of the alkyl chain (from butyl, 0.9, to octyl, 0.7). The L-Val-based amphiphile molecule presented the highest MGCs between 2.9 and > 4.0, while Phe-based ionogel was the most stable (1.4-1.7), likely owing to the presence of the aromatic side chain of L-Phe, which contributes to additional π–π interaction in the self-assembly process.

Palmitoylated amino acid (Gly, L-Val, L-Ile, L-Asp, L-Glu, L-Met, L-Phe, L-Lys)-based ionogelators were developed by Restu and co-workers.^[19]^ This amphiphile molecules were modified at the N-terminus of the amino acid introducing a palmitic acid (Figure 8f). All the amino acid amphiphile molecules developed demonstrated the ability to form gels at low MGC (0.3-1.6 wt.%), in all the fluorinated ILs tested from imidazolium and ammonium-based families. Although palmitoylated methionine (MGC 0.5-1.3 wt%) and palmitoylated phenylalanine (MGC 0.3-1.4 wt%) showed high gelation ability, the difference was not significant when compared with the MGC of the other palmitoylated amino acid molecules (MGC 0.4-1.6 %w/v). This suggests that the nature of amino acids side chain has a negligible impact on the self-assembly mechanism, with any repulsion between the amino acid side chains being minimal in ILs. Additionally, palmitoylated methionine successfully ionogelated the non-fluorinated ILs, [EtMeIm][Gly], [EtMeIm][Ile] at MGC of 2.0 wt% and [EtMeIm][acetate] at MGC 1.9 wt%, while palmitoylated phenylalanine did not.

### 3.4 Other Amino Acid-Based Ionogelators

**Figure 9:**
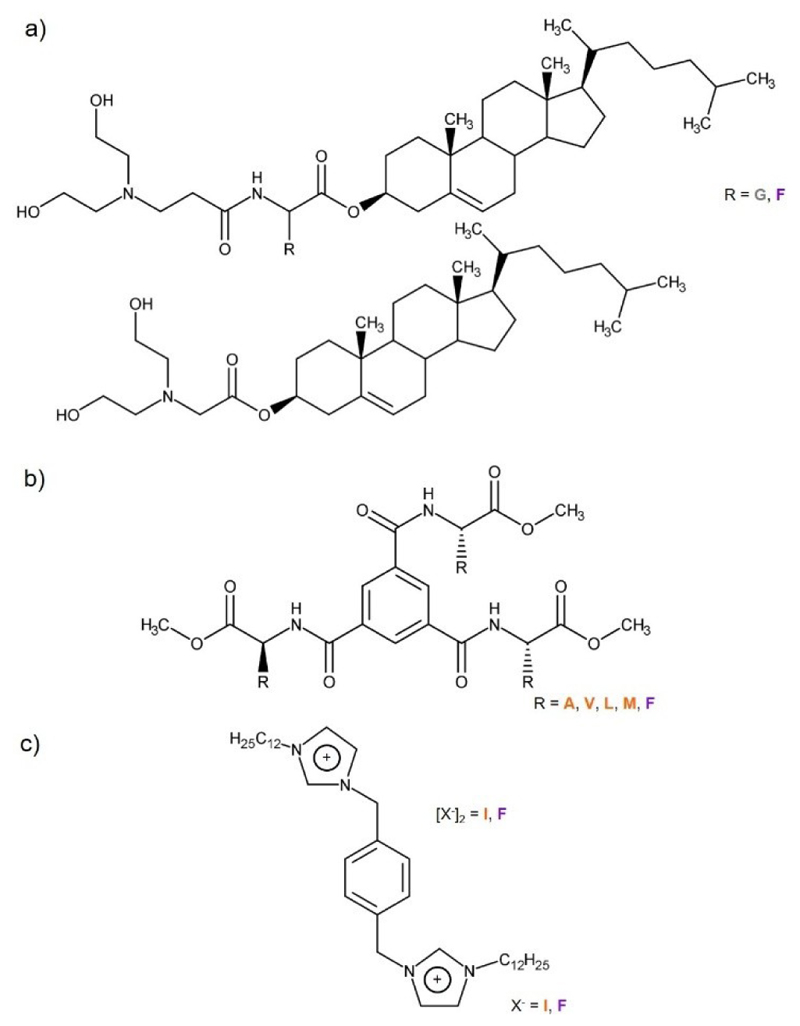
Chemical structures of a) cholesterol-based molecules, b) benzene-tricarboxamides, and c) diimidazolium organic salts as ionogelator molecules. G: glycine. F: phenylalanine. A: alanine. V: valine. L: leucine. M: methionine. I: isoleucine. Amino acid: Aliphatic amino acid. Aromatic amino acid. Other amino acid.

Benzenetricarboxamides,^[16]^ cholesterol-based molecules^[15]^ and diimidazolium organic salts^[18]^ - where the amino acid residue was a substituent or the salt anion - were also investigated as ionogelators (Table 5, Figure 9).

Cholesteryl derivatives were designed by Yan and co-workers.^[15]^ These derivatives comprised a cholesteryl structure and two hydroxyl groups strategically introduced at the end of its hydrophilic part to enhance dissolution in highly polar ILs (Figure 9a). This design aimed to strike a balance between dissolution and precipitation needed to the gel formation. Amino acid residues were selected as spacers. Molecular chirality was identified as a factor influencing the ionogelation ability of cholesteryl-based gelator molecules. The incorporation of the aromatic amino acid D-Phe in the chemical structure proved more effective in gelating ILs from imidazolium and morpholium-based families (0.06-2.0%w/w) compared to L-Phe which was only able to form a gel in the IL [BMMor][I] at MGC of 1.0%w/w. Substituting the aromatic amino acid by the aliphatic amino acid residue Ala, whether L-Ala and D-Ala, resulted in no gel formation, while the substitution with the amino acid residue Gly led to a decrease of ionogelation efficiency, with MGC 2.0-2.5%w/w (also form a gel in the IL [BMPip][TFSA] at MGC 2.5%w/w that the other gelator molecules in the study did not).

Supramolecular ionogelators were developed by Ishioka and co-workers, utilizing 1,3,5-benzenetricarboxylic acid as core unit, modified with methyl esters derived from the aliphatic amino acids L-Ala, L-Val, L-Leu and sulfur-containing L-Met, and the aromatic amino acid L-Phe (Figure 9b).^[16]^ C_3_-symmetrical structure of the core unit, combined with amino acid methyl esters, was anticipated to play a crucial role in facilitating intermolecular hydrogen bonding and π–π interactions, promoting the molecular self-assembly process. Among the gelator molecules, those modified with the aliphatic amino acids (L-Ala, L-Val, L-Leu and L-Met) exhibited superior ionogelation efficiency with MGC of 0.1-1.7 wt% in imidazolium and pyridinium-based ILs compared the gelator containing the aromatic amino acid residue L-Phe which was only able to form ionogels in the imidazolium-based ILs [BuMeIm][TFSA], [EtMeIm][CF_3_SO_3_] and [EtMeIm][PF_6_] at MGC of 1.8-2.0 wt%. This unexpected observation was attributed to the steric hindrance effect exerted by L-Phe residues and the helical-like configuration, characteristic of benzenetricarboxamide structures, which was unfavorable for π–π interactions between aromatic amino acid residues. Gelator molecules containing amino acid Gly showed no gelation ability. Moreover, the IL anion was identified as a potential factor influencing the ionogelation process, as the molecules containing the aliphatic residues L-Val and L-Leu exhibited higher ionogelation abilities towards the IL anion [CF_3_SO_3_]^−^ (MGC 0.1-0.7 wt%), while those containing L-Ala or L-Met showed enhanced ionogelation abilities towards the IL anion [BF_4_]^−^ (MGC 0.5-0.7 wt%).

A slightly different approach in designing successful ionogelator molecules was presented by Rizzo and co-workers. In this specific study, amino acids served as the anions in diimidazolium-based organic salts gelator molecules (Figure 9c).^[18]^ The aliphatic amino acid-based salt, [*p*-C12im][L-Ile]_2_ demonstrated superior ionogelation ability compared to the aromatic amino acid-based salt [*p*-C12im][L-Phe]_2_, forming an ionogel in both hydrophilic ILs tested, [BMIm][BF_4_] and [BMIm][PF_6_]. Nonetheless, the influence of the IL anion was clear, as the MGC significantly increased for the ionogelation in [BMIm][BF_4_] (2.3 *vs* 6.3 w/w%). The [BF_4_]^−^ anion, being a slightly stronger hydrogen-bond acceptor than [PF_6_]^−^ anion according to empirical Kamlet-Taff parameters (with a hydrogen bond acceptor ability (β) value of 0.55 for [BF_4_]^−^ and 0.44 for [PF_6_]^−[38]^), played a crucial role in this effect. Notably, [*p*-C12im][L-Phe]_2_ could only form a gel in [BMIm][BF_4_] at similar MGC that, in this specific case, the nature of the amino acid used as organic salt anion did not significantly influenced process self-assembly.

## 4 Summary and Outlook

Supramolecular self-assembly in non-conventional solvents, as ILs and DESs, presents inherent challenges, often relying on serendipitous processes driven by the diverse and complex nature of these solvents.

Peptides and amino acids are the perfect building blocks for self-assembly processes due to their chemical diversity. This diversity arises from the unique structural and functional characteristics of different amino acids. The variability in side chains among these building blocks results in differences in polarity, charge, and hydrophobicity. The existence of chirality, using L- and/or D-amino acids, and other chemical modifications further enhance the chemical diversity of these building blocks.

In the last years, peptide and amino acid-based gelators have been employed for eutectogels and ionogels.

Typical non-covalent cross-linking strategies employed in the formation of the biological scaffold in ILs concerned H-bonding and coulombic interactions, with ionogelator content as low as 0.06-10 wt%.

The self-assembly process in DESs generating eutectogels, typical concerned H-bonding conjugated with solvophobic effects. Biological scaffold content in eutectogelators (0.5-10 wt %) as revealed to be similar as ionogelators. Moreover, only choline chloride-based DESs have been employed in the exploitation of peptide and amino acid-based as eutectogelators. Concerning the wide diversity of DES chemistry, many other DESs can be explored in the future.

The rational design of peptide and amino acid-based ionogels and eutectogels requires a multidisciplinary approach spanning from chemistry to materials science and bioengineering. A key requirement is the existence of stronger non-covalent self-interactions within the gelator molecules than with the solvent molecules of the surrounding environment. Nonetheless, these self-interactions must not be so strong as to lead to precipitation of the gelator molecules within the IL or DES. A fine hydrophilic-lipophilic balance would be crucial for the supramolecular self-assembly process.

The self-organization of peptides and amino acid in non-conventional solvents, as ILs or DESs, engender innovative, biocompatible and biodegradable soft-materials with unique properties and functionalities with wide application fileds, namely in biomedicine and wearable bioelectronics.

## Figures and Tables

**Table 1:** Short peptide ionogelators and dipeptide amphiphile eutectogelators.

Gelator		Non-conventional solvent	Ref.
cyclo(L-*β*-3,7-dimethyloctylasparaginyl-L-phenylalanyl) cyclo(L- *β*-2-ethylhexylasparaginyl-L-phenylalanyl)	ILs	Imidazolium, pyridinium, piperidinium and ammonium-based ILs ([EMIm][BF_4_], [EMIm][TFSA], [PMIm][BF_4_], [PMIm] [TFSA], [BMIm][BF_4_], [BMIm][PF_6_], [BMIm][TFSA], [BMIm][CF_3_SO_3_], [BMIm][-ClO_4_], [HMIm][BF_4_], [HMIm][PF_6_], [HMIm][I], [BPy][BF_4_], [HPy][TFSA], [HMPy][TFSA], [BMPyr][TFSA], [BMPip][TFSA], [HMMor][TFSA], [TMHAmmo][TFSA], [MTOAm-mo][TFSA], [DEMEMAm-mo] [BF_4_], [DEMEMAmmo] [TFSA], [DEHEEMAmmo] [BF_4_], [DEHEEMAmmo][TFSA])	^[6]^
Phe-Phe-based amphiphile molecule Trp-Phe-based amphiphile molecule Ala-Ala-based amphiphile molecule	DESs (MR)	Choline chloride:phenyl-acetic acid (1 : 2)	^[7]^

Phe: phenylalanine. Trp: tryptophan. Ala: alanine. ILs; Ionic Liquids. DESs: Deep eutectic solvents. MR: molar ratio. [EMIm]: 1-ethyl-3-methylimidazolium. [PMIm]: 1-propyl-3-methylimidazolium. [BMIm]: 1-butyl-3-methylimidazolium. [HMIm]: 1-hexyl-3-methylimidazolium. [BPy]: 1-butylpyridinium. [HPy]: 1-hexylpyridinium. [HMPy]: 1-hexyl-4-methylpyridinium. [BMPyr]: 1-butyl-1methylpyrrolidinium. [BMPip]: 1-butyl-1-methylpiperidinium. [HMMor]: 1-hexyl-1-methylmorpholinium. [TMHAmmo]: trimethylhexylammonium. [MTOAmmo]: methyltrioctylammonium. [DEMEMAmmo]: diethyl(2-methoxyethyl)methylammonium. [DEHEEMAmmo]: diethyl(2-(2-hydroxyethoxy)ethyl)methylammonium. [BF_4_]: tetrafluoroborate. [TFSA]: bis(trifluoromethylsulfonyl)imide. [PF_6_]: hexafluorophosphate. [CF_3_SO_3_]: trifluoromethanesulfonate. [ClO_4_]: perchlorate. [I]: iodide.

**Table 2:** Amino acid eutectogelators.

Gelator	Deep eutectic solvent (MR)	Ref.
L-Trp	Choline chloride:urea (1 : 2)	^[10]^
	Choline chloride:phenylacetic acid (1 : 2)	^[9],[8]^
L-Ile	Choline chloride:phenylacetic acid (1 : 2)	^[9],[8]^
L-Phe	Choline chloride:phenylacetic acid (1 : 2)	^[9]^
L-Leu	Choline chloride:phenylacetic acid (1 : 2)	^[9]^
L-Pro	Choline chloride:urea (1 : 2)	^[10]^
	Choline chloride:phenylacetic acid (1 : 2)	^[9]^
L-Ser L-Pro-NH_2_ t-4-OH-L-Pro	Choline chloride :urea (1 : 2)	^[10]^

MR: molar ratio. Trp: tryptophan. Ile: isoleucine. Phe: phenylalanine. Leu: leucine. Pro: proline. Ser: serine. L-Pro-NH_2_: L-prolinamide. t-4-OH−L-Pro: *trans*-4-hydroxy-L-proline.

**Table 3:** Protected amino acid eutectogelators.

Gelator	Deep eutectic solvent (MR)	Ref.
Fmoc-Phe	Choline chloride:phenylacetic acid (1 : 2)	^[9]^
	Choline chloride:malonic acid (2 : 2)	^[11]^
Fmoc-L–Ile	Choline chloride:phenylacetic acid (1 : 2)	^[9]^
Fmoc-HomoPhe	Choline chloride:malonic acid (2 : 2)	^[11]^
Fmoc-Leu		
Fmoc-Val		
Fmoc-Ala	Choline chloride:malonic acid (2 : 2)	^[11]^
	Choline chloride:oxalic acid (2 : 2)	^[11]^
	Choline chloride:glycerol (2 : 4)	^[11]^

MR: molar ratio. Trp: tryptophan. Phe: phenylalanine. Ile: isoleucine. Leu: leucine. Pro: proline. Ser: serine. Ala: alanine. Asp: Asparticine. Val: valine. Fmoc: fluorenylmethoxycarbonyl.

**Table 4:** Amino acid-based amphiphiles.

Gelator		Non-conventional solvents	Ref.
N-octadecyl benzyloxy(carbonyl)-L-phenylalanine benzyloxy(carbonyl)-L-phenylalanine derived	IL	Trihexyl (tetradecyl)phosphonium bis(trifluoromethylsulfonyl)imide ([P_6,6,6,14_][TFSA])	^[20]^
Palmitoylated amino acids (Gly, L-Val, L-Ile, L-Asp, L-Glu, L-Met, L-Phe, L-Lys)	IL	Imidazolium and ammonium-based ILs ([BMIm][TFSA], [EMIm][TFSA], [BMIm][CF_3_SO_3_], [EMIm][CF_3_SO_3_], [TMHA][TFSA])	^[19]^
Amino acid-derive urea molecules (Gly, L-Ala, L-Val, L-Ile, L-Phe)	IL	Imidazolium-based IL ([MBIm][PF_6_], [MHIm][PF_6_], [MOIm][PF_6_], [MBIm][BF_4_], [MBIm][TFSA], [MOIm][TF-SA])	^[17]^
Cholesterol-based molecules (aa as spacer – L-Phe, D-Phe, Gly)	IL	Imidazolium, pyridinium, piperidinium and morpholinium-based ILs ([BMIm][BF_4_], [HMIm][BF_4_], [HEMIm][BF_4_], [BMIm][TFSA], [BMMor][I], [BMPy][TF-SA])	^[15]^
Amino acid-based amphiphile molecules (L-Gly, L-Val, L-Leu, L-Ile, L-Phe, D-Val)	IL	Imidazolium, pyridinium, piperidinium ILs ([EMIm][TFSA], [BMIm][TFSA], [HMIm][TFSA], [BuPy][TFSA], [BMIm][CF_3_SO_3_], [MTBAmmo][TFSA], [BMPi][TFSA])	^[14]^
Amino acid-based amphiphile molecules (L-Trp, L-Phe, L-Alal)	DES (MR)	Choline chloride:phenylacetic acid (1 : 2) Choline chloride:levulinic acid (1 : 2)	^[7]^
Amino acid-based amphiphile molecules (L-Trp, L-Phe, L–Ile, L–Val)	IL	Imidazolium and pyridinium-based ILs ([BMIm][Br] 10 %aq, [BMIm][Cl] 10 %aq, [BMIm][BF_4_], [BMIm][PF_6_], [BPy][Br] 10 % aq, [BPy][BF_4_], [BMPy][BF_4_])	^[13]^

Gly: glycine. Val: valine. Ile: isoleucine. Asp: aspartic acid. Glu: glutamic acid. Met: metionine. Phe: phenylalanine. Lys: lysine. Leu: leucine. IL: ionic liquid. DES: deep eutectic solvent. MR: molar ratio. [EMIm]: 1-ethyl-3-methylimidazolium. [BMIm]: 1-butyl-3-methylimidazolium. [HMIm]: 1-hexyl-3-methylimidazolium. [BPy]: 1-butylpyridinium. [HPy]: 1-hexylpyridinium. [HMPy]: 1-hexyl-4-methylpyridinium. [BMPyr]: 1-butyl-1methylpyrrolidinium. [BMPip]: 1-butyl-1-methylpiperidinium. [HMMor]: 1-hexyl-1-methylmorpholinium. [TMHAmmo]: trimethylhexylammonium. [MTBAmmo]: methyltributylammonium. [BF_4_]: tetrafluoroborate. [TFSA]: bis(trifluoromethylsulfonyl)imide. [PF_6_]: hexafluorophosphate. [CF_3_SO_3_]: trifluoromethanesulfonate. [Cl]: chloride. [Br]: bromide. aq: aqueous.

**Table 5:** Amino acid-based molecules leading to ionogelation.

Gelator	Ionic liquids	Ref.
Cholesterol-based molecules (aa as spacer – L-Phe, D-Phe, Gly)	Imidazolium, pyridinium, piperidinium and morpholinium-based ILs ([BMIm][BF_4_], [HMIm][BF_4_], [C_2_OHMIM][BF_4_], [BMIm][TFSA], [BMMor][I], [BMPip][TFSA])	^[15]^
1,3,5-benzenetricarboxylic acids modified with amino acid methyl esters (L-Ala, L-Val, L-Leu, L-Met, L-Phe)	Imidazolium, pyridinium and ammonium-based ILs ([EMIm][TFSA], [BMIm][TFSA], [HMIm][TFSA], [BuPy][TFSA], [TMPAmmo][TFSA], [EMIm][CF_3_SO_3_], [BMIm][CF_3_SO_3_], [EMIm][BF_4_], [BMIm][BF_4_], [EtMeIm][PF_6_])	^[16]^
Diimidazolium-based organic salts, bearing amino acids as anions (L-Ile, L-Phe) Cation: [*p*-C_12_im]	Imidazolium-based IL ([BMIm][PF_6_], [BMIm][BF_4_])	^[18]^

Gly: glycine. Val: valine. Ile: isoleucine. Met: metionine. Phe: phenylalanine. Leu: leucine. IL: ionic liquid. [EMIm]: 1-ethyl-3-methylimidazolium. [BMIm]: 1-butyl-3-methylimidazolium. [HMIm]: 1-hexyl-3-methylimidazolium. [C_2_OHMIM]: 1-carboxymethyl-3-methylimidazolium. [BPy]: 1-butylpyridinium. [HPy]: 1-hexylpyridinium. [BMPip]: 1.–butyl-1methylpiperidinium. [TMPAmmo]: trimethylpropylammonium. [BF_4_]: tetrafluoroborate. [TFSA]: bis(trifluoromethylsulfonyl)imide. [PF_6_]: hexafluorophosphate. [CF_3_SO_3_]: trifluoromethanesulfonate.

## Data Availability

Data sharing is not applicable to this article as no new data were created or analyzed in this study.
